# Supplementary material for: Verbal manipulations of learning expectancy do not enhance reconsolidation
Source: PLoS One. 2022 Aug 18;17(8):e0241943. doi: 10.1371/journal.pone.0241943 (PMC9387781; doi:10.1371/journal.pone.0241943)
Supplement: S1 Data — (DOCX) [file pone.0241943.s001.docx]

Supplementary Data

*Day 1 acquisition (EDA)*

|  | Expectation for learning group | | | No expectation for learning group | | | No reactivation | | |
| --- | --- | --- | --- | --- | --- | --- | --- | --- | --- |
| Trial | CS+ (SD) | CS- (SD) | CS+US (SD) | CS+ (SD) | CS- (SD) | CS+US (SD) | CS+ (SD) | CS- (SD) | CS+US (SD) |
| 1 | 0.975 (0.607) | 0.702 (0.563) | 0.959 (0.630) | 0.889 (0.992) | 1.159 (0.981) | 0.889 (0.992) | 0.808 (1.051) | 0.601 (0.662) | 0.808 (1.051) |
| 2 | 0.534 (0.387) | 0.602 (0.567) | 0.512 (0.500) | 0.948 (0.977) | 0.747 (0.822) | 0.512 (0.498) | 0.671 (0.577) | 0.587 (0.695) | 0.433 (0.439) |
| 3 | 0.593 (0.502) | 0.503 (0.492) | 0.424 (0.342) | 0.485 (0.512) | 0.773 (0.937) | 0.933 (0.899) | 0.438 (0.443) | 0.635 (0.604) | 0.368 (0.459) |
| 4 | 0.401 (0.461) | 0.425 (0.532) | 0.447 (0.476) | 0.939 (0.896) | 0.704 (0.700) | 0.513 (0.484) | 0.504 (0.524) | 0.532 (0.434) | 0.495 (0.442) |
| 5 | 0.492 (0.396) | 0.443 (0.366) | 0.484 (0.554) | 0.757 (0.910) | 0.315 (0.513) | 0.703 (1.355) | 0.609 (0.607) | 0.313 (0.368) | 0.444 (0.491) |
| 6 | 0.468 (0.463) | 0.311 (0.372) | 0.461 (0.638) | 0.487 (0.528) | 0.591 (1.089) | 0.981 (1.054) | 0.451 (0.490) | 0.422 (0.576) | 0.474 (0.512) |
| 7 | 0.461 (0.561) | 0.396 (0.455) | - | 0.803 (1.413) | 0.483 (0.597) | - | 0.496 (0.524) | 0.226 (0.246) | - |
| 8 | 0.451 (0.644) | 0.120 (0.308) | - | 0.935 (1.090) | 0.419 (0.661) | - | 0.335 (0.374) | 0.205 (0.277) | - |

*Note.* Aggregate means for skin conductance response (EDA [µS]) in each group. Values are untransformed. SD = standard deviation.

*Day 2 reactivation and extinction (EDA)*

|  | Expectation for learning group | | No expectation for learning group | | No reactivation | |
| --- | --- | --- | --- | --- | --- | --- |
| Trial | CS+ (SD) | CS- (SD) | CS+ (SD) | CS- (SD) | CS+ (SD) | CS- (SD) |
| 1 | *0.947 (1.335) | 1.026 (0.911) | *0.947 (1.335) | 0.920 (0.956) | 1.229 (1.441) | 0.706 (0.721) |
| 2 | 1.110 (0.883) | 0.853 (1.044) | 1.110 (0.883) | 0.255 (0.360) | 0.702 (0.808) | 0.718 (1.055) |
| 3 | 0.748 (0.678) | 0.383 (0.396) | 0.748 (0.678) | 0.587 (1.250) | 0.685 (0.933) | 0.397 (0.517) |
| 4 | 0.386 (0.678) | 0.566 (0.978) | 0.386 (0.678) | 0.156 (0.211) | 0.462 (0.735) | 0.312 (0.524) |
| 5 | 0.176 (0.335) | 0.525 (0.690) | 0.176 (0.335) | 0.143 (0.426) | 0.417 (0.642) | 0.250 (0.518) |
| 6 | 0.107 (0.164) | 0.316 (0.619) | 0.107 (0.164) | 0.165 (0.308) | 0.279 (0.482) | 0.139 (0.278) |
| 7 | 0.405 (0.728) | 0.333 (0.730) | 0.405 (0.728) | 0.067 (0.160) | 0.211 (0.395) | 0.074 (0.155) |
| 8 | 0.082 (0.136) | 0.156 (0.336) | 0.082 (0.136) | 0.256 (0.895) | 0.123 (0.234) | 0.077 (0.155) |
| 9 | 0.278 (0.491) | 0.252 (0.713) | 0.278 (0.491) | 0.227 (0.666) | 0.181 (0.320) | 0.032 (0.081) |
| 10 | 0.272 (0.513) | 0.176 (0.284) | 0.272 (0.513) | 0.108 (0.190) | 0.329 (0.622) | 0.153 (0.252) |
| 11 | 0.296 (0.600) | 0.057 (0.180) | 0.296 (0.600) | 0.318 (0.760) | 0.191 (0.484) | 0.295 (0.647) |

*Note.* Aggregate means for skin conductance response (EDA [µS]) in each group. Values are untransformed. SD = standard deviation. *Reactivation trial

*Day 3 reinstatement (EDA)*

|  | Expectation for learning group | | No expectation for learning group | | No reactivation | |
| --- | --- | --- | --- | --- | --- | --- |
| Trial | CS+ (SD) | CS- (SD) | CS+ (SD) | CS- (SD) | CS+ (SD) | CS- (SD) |
| 1 | 1.066 (1.130) | 1.201 (1.289) | 1.002 (1.386) | 1.142 (1.296) | 0.782 (0.968) | 0.614 (0.641) |
| 2 | 0.254 (0.691) | 0.728 (0.962) | 0.775 (0.698) | 0.914 (1.787) | 0.382 (0.468) | 0.413 (0.504) |
| 3 | 0.215 (0.436) | 0.197 (0.485) | 0.745 (1.262) | 0.261 (0.678) | 0.168 (0.233) | 0.315 (0.442) |
| 4 | 0.151 (0.332) | 0.202 (0.296) | 0.326 (0.446) | 0135 (0.291) | 0.294 (0.598) | 0.112 (0.217) |
| 5 | 0.118 (0.332) | 0.148 (0.267) | 0.219 (0.727) | 0.300 (0.701) | 0.056 (0.204) | 0.366 (1.107) |
| 6 | 0.170 (0.415) | 0.951 (1.560) | 0.158 (0.255) | 0.539 (1.147) | 0.102 (0.189) | 0.175 (0.519) |
| 7 | 0.033 (0.075) | 0.072 (0.150) | 0.158 (0.298) | 0.220 (0.500) | 0.181 (0.328) | 0.089 (0.257) |
| 8 | 0.472 (1.269) | 0.374 (0.712) | 0.180 (0.431) | 0.412 (1.061) | 0.017 (0.060) | 0.170 (0.328) |
| 9 | 0.077 (0.138) | 0.146 (0.342) | 0.539 (0.903) | 0.242 (0.461) | 0.186 (0.482) | 0.314 (0.780) |
| 10 | 0.164 (0.265) | 0.419 (0.926) | 0.413 (0.892) | 0.273 (0.858) | 0.138 (0.347) | 0.113 (0.271) |

*Note.* Aggregate means for skin conductance response (EDA [µS]) in each group. Values are untransformed. SD = standard deviation.

*Day 1 acquisition (FPS)*

|  | Expectation for learning group | | | No expectation for learning group | | | No reactivation | | |
| --- | --- | --- | --- | --- | --- | --- | --- | --- | --- |
| Trial | CS+ (SD) | CS- (SD) | CS+US (SD) | CS+ (SD) | CS- (SD) | CS+US (SD) | CS+ (SD) | CS- (SD) | CS+US (SD) |
| 1 | 29.460 (17.143) | 30.441 (25.420) | 29.460 (17.143) | 31.528 (12.000) | 30.629 (17.746) | 31.528 (12.000) | 33.652 (19.569) | 37.805 (25.334) | 33.652 (19.569) |
| 2 | 31.756 (22.569) | 29.964 (20.85) | 26.295 (21.890) | 31.097 (16.669) | 28.430 (22.816) | 23.940 (16.157) | 29.005 (19.048) | 24.973 (17.569) | 29.566 (22.476) |
| 3 | 26.300 (17.966) | 27.700 (23.935) | 22.479 (16.181) | 26.499 (16.910) | 28.568 (16.986) | 29.488 (20.549) | 29.644 (22.055) | 22.431 (16.462) | 26.106 (17.768) |
| 4 | 24.164 (20.951) | 20.916 (11.764) | 24.286 (26.471) | 29.184 (20.700) | 25.147 (19.215) | 25.768 (19.710) | 28.576 (19.809) | 29.045 (20.546) | 30.948 (22.319) |
| 5 | 23.249 (18.335) | 23.739 (16.501) | 17.930 (11.429) | 27.505 (16.544) | 23.779 (18.122) | 27.061 (16.465) | 30.256 (22.975) | 27.159 (22.189) | 25.322 (20.985) |
| 6 | 24.110 (25.362) | 21.404 (13.901) | 23.366 (17.896) | 26.715 (19.476) | 21.880 (13.608) | 25.946 (17.626) | 27.731 (22.915) | 25.919 (25.364) | 24.387 (22.923) |
| 7 | 18.632 (12.218) | 18.835 (12.991) | - | 26.921 (15.783) | 19.609 (13.330) | - | 25.609 (21.474) | 27.851 (22.144) | - |
| 8 | 23.658 (17.655) | 20.745 (21.973) | - | 25.933 (17.789) | 21.050 (18.037) | - | 24.728 (22.241) | 22.429 (20.432) | - |

*Note.* Aggregate means for fear potentiated startle (FPS [µV]) in each group. Values are untransformed. SD = standard deviation.

*Day 2 reactivation and extinction (FPS)*

|  | Expectation for learning group | | No expectation for learning group | | No reactivation | |
| --- | --- | --- | --- | --- | --- | --- |
| Trial | CS+ (SD) | CS- (SD) | CS+ (SD) | CS- (SD) | CS+ (SD) | CS- (SD) |
| 1 | - | 37.955 (38.418) | - | 29.974 (26.251) | 37.343 (30.008) | 34.517 (28.876) |
| 2 | 30.589 (21.900) | 29.566 (35.629) | 38.258 (24.430) | 27.641 (24.759) | 36.231 (30.543) | 30.022 (23.771) |
| 3 | 35.130 (37.874) | 33.292 (39.552) | 29.034 (24.817) | 30.527 (24.168) | 29.212 (22.935) | 29.366 (27.612) |
| 4 | 27.987 (34.844) | 32.642 (41.766) | 27.306 (22.567) | 26.096 (21.647) | 24.440 (20.949) | 25.060 (23.305) |
| 5 | 29.514 (36.868) | 27.324 (38.876) | 24.427 (21.502) | 21.030 (22.446) | 27.444 (24.622) | 23.987 (23.464) |
| 6 | 32.732 (40.795) | 29.214 (39.619) | 27.602 (21.613) | 22.584 (22.466) | 22.457 (18.023) | 25.526 (26.634) |
| 7 | 28.379 (37.463) | 17.718 (13.984) | 20.457 (21.376) | 23.431 (20.686) | 25.171 (23.888) | 23.024 (20.837) |
| 8 | 25.823 (33.491) | 18.299 (17.964) | 27.486 (26.936) | 20.593 (20.857) | 21.213 (16.404) | 19.501 (20.950) |
| 9 | 29.992 (38.173) | 26.590 (39.782) | 23.518 (21.400) | 20.477 (20.187) | 20.785 (18.983) | 21.022 (22.355) |
| 10 | 19.059 (15.588) | 27.669 (39.545) | 25.846 (21.996) | 20.495 (20.330) | 23.480 (21.935) | 20.941 (20.053) |
| 11 | 25.908 (38.690) | 28.089 (39.023) | 23.986 (23.034) | 17.888 (19.518) | 20.434 (18.556) | 20.684 (22.712) |

*Note.* Aggregate means for fear potentiated startle (FPS [µV]) in each group. Values are untransformed. SD = standard deviation. FPS was not measured for the reactivation trial (CS+ trial 1) in the expectation for learning and no expectation for learning groups.

*Day 3 reinstatement (FPS)*

|  | Expectation for learning group | | No expectation for learning group | | No reactivation | |
| --- | --- | --- | --- | --- | --- | --- |
| Trial | CS+ (SD) | CS- (SD) | CS+ (SD) | CS- (SD) | CS+ (SD) | CS- (SD) |
| 1 | 27.657 (19.459) | 30.734 (23.491) | 40.049 (23.632) | 36.638 (20.477) | 33.027 (28.239) | 24.569 (17.804) |
| 2 | 21.050 (20.221) | 30.784 (23.625) | 31.541 (21.672) | 33.302 (25.072) | 32.306 (26.555) | 23.390 (17.045) |
| 3 | 21.920 (17.575) | 25.953 (21.180) | 28.272 (23.267) | 28.876 (21.100) | 25.920 (21.426) | 20.214 (16.686) |
| 4 | 17.592 (15.042) | 21.761 (18.033) | 23.589 (17.996) | 26.365 (19.221) | 20.290 (17.622) | 18.142 (12.462) |
| 5 | 15.699 (16.538) | 17.032 (16.268) | 28.705 (21.133) | 21.971 (16.685) | 21.321 (20.027) | 18.639 (16.307) |
| 6 | 15.959 (15.954) | 19.493 (18.672) | 17.673 (13.577) | 22.706 (18.749) | 21.112 (19.018) | 19.336 (18.479) |
| 7 | 17.288 (16.515) | 17.811 (19.220) | 22.224 (20.294) | 19.588 (16.378) | 14.967 (13.738) | 16.449 (14.351) |
| 8 | 18.596 (16.987) | 17.153 (18.546) | 14.967 (9.235) | 20.538 (16.900) | 18.653 (22.273) | 19.438 (19.914) |
| 9 | 13.507 (11.159) | 15.431 (18.002) | 20.406 (18.169) | 16.760 (15.807) | 19.407 (18.525) | 17.677 (15.143) |
| 10 | 16.809 (18.923) | 15.516 (20.554) | 19.726 (18.374) | 17.961 (18.853) | 16.537 (15.234) | 18.010 (16.164) |

*Note.* Aggregate means for fear potentiated startle (FPS [µV]) in each group. Values are untransformed. SD = standard deviation.
