# Supplementary material for: Verbal manipulations of learning expectancy do not enhance reconsolidation
Source: PLoS One. 2022 Aug 18;17(8):e0241943. doi: 10.1371/journal.pone.0241943 (PMC9387781; doi:10.1371/journal.pone.0241943)
Supplement: Attachment — Submitted filename: Reply to Reviewers-Final.docx [file pone.0241943.s003.docx]

Comments to the Authors

**Comments to the Author**

1. If the authors have adequately addressed your comments raised in a previous round of review and you feel that this manuscript is now acceptable for publication, you may indicate that here to bypass the “Comments to the Author” section, enter your conflict of interest statement in the “Confidential to Editor” section, and submit your "Accept" recommendation.

Reviewer #3: (No Response)

Reviewer #4: (No Response)

Reviewer #5: (No Response)

2. Is the manuscript technically sound, and do the data support the conclusions?

The manuscript must describe a technically sound piece of scientific research with data that supports the conclusions. Experiments must have been conducted rigorously, with appropriate controls, replication, and sample sizes. The conclusions must be drawn appropriately based on the data presented.

Reviewer #3: Yes

Reviewer #4: No

Reviewer #5: Partly

3. Has the statistical analysis been performed appropriately and rigorously?

Reviewer #3: Yes

Reviewer #4: Yes

Reviewer #5: Yes

4. Have the authors made all data underlying the findings in their manuscript fully available?

The [PLOS Data policy](http://www.plosone.org/static/policies.action#sharing) requires authors to make all data underlying the findings described in their manuscript fully available without restriction, with rare exception (please refer to the Data Availability Statement in the manuscript PDF file). The data should be provided as part of the manuscript or its supporting information, or deposited to a public repository. For example, in addition to summary statistics, the data points behind means, medians and variance measures should be available. If there are restrictions on publicly sharing data—e.g. participant privacy or use of data from a third party—those must be specified.

Reviewer #3: No

Reviewer #4: No

Reviewer #5: No

*We have added supplementary data with aggregate means.*

5. Is the manuscript presented in an intelligible fashion and written in standard English?

PLOS ONE does not copyedit accepted manuscripts, so the language in submitted articles must be clear, correct, and unambiguous. Any typographical or grammatical errors should be corrected at revision, so please note any specific errors here.

Reviewer #3: Yes

Reviewer #4: Yes

Reviewer #5: No

6. Review Comments to the Author

Please use the space provided to explain your answers to the questions above. You may also include additional comments for the author, including concerns about dual publication, research ethics, or publication ethics. (Please upload your review as an attachment if it exceeds 20,000 characters)

Reviewer #3: In the present study entitled ‘Impact of the expectation on memory reconsolidation using a post retrieval extinction paradigm’, by Marinos and Ashbaugh, authors aimed to investigate the effects of the expectation for learning on reconsolidation of conditioned fear memories using the post-retrieval extinction paradigm in humans. For this purpose, 48 healthy participants underwent a fear conditioning experiment that took place over three consecutive days. The expectation for learning was manipulated giving instructions prior to memory reactivation; fear potentiated startle (FPS) and skin conductance response (SCR) were taken as implicit measures of fear. Results showed a small effect of the expectation for learning on reconsolidation with FPS as a measure of fear, but no evidence of reconsolidation was observed for SCR. Authors conclude stating that a verbal manipulation of the expectation for learning may not be salient enough to induce reconsolidation as measured by SCR but may be sufficient as measured by FPS.
In general, I think the idea of this article is interesting and the authors’ fascinating observations may be of interest to the readers of Plos One. However, some comments, as well as some crucial citations that should be included to support the authors’ argumentation, need to be addressed to improve the article, its adequacy, and its readability prior to the publication in the present form.

Comments
• Regarding the Abstract: I think that the lack of an explanation of what “expectation for learning” means in this study makes the reader unable to grasp the key aspects of this paper by consulting the abstract. I suggest reorganizing the abstract, making sure to include an explanation of this concept. Also, keywords are missing.

*We have revised the abstract to provide a clearer explanation of what expectation for learning means (see page 2), within the scope of the word count limit of the journal.*

• Regarding the Introduction: In general, I strongly recommend authors to reshape this section, which I believe is way too much simplistic to outline a comprehensive yet rigorous investigation of the subject of this study (i.e., memory reconsolidation). Thus, I suggest the authors to make such effort to provide a brief overview of the pertinent published literature that offer a more in depth as well scientific perspective on reconsolidation, to provide a more defined background.

*We thank the reviewer for this comment. We have revised the introduction to include a more in-depth overview of reconsolidation. For example, on page 4 and 5, we have included more recent studies that use various techniques to manipulate reconsolidation, such as transcranial magnetic stimulation. Based upon feedback in subsequent comments we have included the following references:*

1. *Agren T. Human reconsolidation: A reactivation and update. Brain Research Bulletin. 2014;105:70-82. doi:10.1016/j.brainresbull.2013.12.010.*
2. *Borgomaneri S, Battaglia S, Garofalo S, Tortora F, Avenanti A, di Pellegrino G. State-dependent TMS over prefrontal cortex disrupts fear-memory reconsolidation and prevents the return of fear. Curr Biol. 2020;30(18):3672-3679. doi: 10.1016/j.cub.2020.06.091.*
3. *Borgomaneri S, Battaglia S, Sciamanna G, Tortora F, Laricchiuta D. Memories are not written in stone: Re-writing fear memories by means of non-invasive brain stimulation and optogenetic manipulations. Neurosci Biobehav Rev. 2021;127:334-352.* [*https://doi.org/10.1016/j.neubiorev.2021.04.036*](https://doi.org/10.1016/j.neubiorev.2021.04.036)*.*
4. *Borgomaneri S, Battaglia S, Avenanti A, di Pellegrino G. Don't hurt me no more: State-dependent transcranial magnetic stimulation for the treatment of specific phobia. J. Affect. Disord. 2021;286:78.79. https://doi.org/10.1016/j.jad.2021.02.076.*

• Page 2: correct a typo, change “enhanced” in “enhance”.

*We have replaced “enhances” with “enhanced” (see page 2).*

• Page 3-6, Introduction: Authors provided a detailed overview on the different methods used to interfere with the reconsolidation process, only focusing on pharmacological interventions. However, the literature they cite is neither exhaustive nor up to date. Still, I think that adding some studies that could provide further insight on different techniques (i.e., Non-invasive brain stimulation techniques, NIBS) used to study reconsolidation would be crucial in this section: for example, Borgomaneri and colleagues’ study (2020, Current Biology - https://doi.org/10.1016/j.cub.2020.06.091) showed that the inhibition with of the dorsolateral prefrontal cortex (DLPFC) with Transcranial magnetic stimulation (TMS) after emotional memory reactivation disrupts physiological responding to learned fear, highlighting the role of this area in the neural network that mediates the reconsolidation of fear memories in humans. I also suggest mentioning a review by Borgomaneri and colleagues (2021, Neuroscience and Biobehavioral Reviews - https://doi.org/10.1016/j.neubiorev.2021.04.036) that specifically focused on human reconsolidation and on the ability of non-invasive brain simulation (NIBS) to interfere with activity of neural circuits (i.e., amygdala-mPFC-hippocampus) involved in the acquisition and reconsolidation of emotional memories. Finally, I would suggest one of the latest Borgomaneri and colleagues’ study (2021, Journal of Affective Disorders - https://doi.org/10.1016/j.jad.2021.02.076), that illustrated the therapeutic potential of NIBS as a valid alternative in the treatment of abnormally persistent memories that characterized those patients with anxiety disorders that do not respond to psychotherapy and/or drug treatments, promoting reduction of fear by focusing on the reconsolidation process. Moreover, if the authors consider it appropriate, they can also see additional studies, for example Censor and colleagues’ study (2014, Cortex - https://doi.org/10.1016/j.cortex.2013.05.013) or Sandrini and colleagues’ study (2018, Frontiers in psychology – https://doi.org/10.3389/fpsyg.2018.01430) that provide a general overview on the topic.

*We thank you for these suggestions and have included the above references in the introduction on pages 5 and 6.*

• Page 4: please add “to” after “translational impact”.

*We have added the word “to” to this sentence (see page 4).*

• Page 4: correct a typo, change “sufficient” to “sufficient”.

*We have corrected this typo (see page 5).*

• Page 5: correct a typo, change “propanrolol” to “propranolol”.

*We thank the reviewer for their careful attention to detail. We did a find and replace search for “propanrolol” and were unable to locate the typo. We are happy to include line numbers (if permitted by the journal) to assist in locating that typo.*

• Page 6: The hypothesis of the study is clearly presented and adequately outlined; however, authors should consider adding a detailed definition of what the expectation of learning, as it is the variable that is manipulated in the study.

*We have added a definition for expectation for learning on page 6: The expectation for learning refers to anticipation that new learning about a contingency is going to occur.*

• Page 9: correct a typo, change “participates” in “participants”.

*This typo has been corrected (see page 9).*

• Page 8, Unconditioned stimulus (US): please provide the mean shock intensity for each group.

*Please note that this is reported in table 1.*

• Page 10: specify the acronyms “FPS” after the full name “Fear potentiated startle”.

*The acronym FPS was added after the full name (see page 11).*

• Regarding the Methods: What were the criteria adopted for usable physiological data? Authors stated that 12 participants were excluded because their physiological data was not readable. Please explain in detail what is meaning ‘not readable’ and use the proper study to exclude data from the dataset collected.

*We have further clarified what we mean be the data of those 12 participants not being readable on page 9:*

*12 were excluded because their physiological data were excessively noisy overall based on visual inspection (e.g., due to poor electrode contact) or evidence of clipping (e.g., data that were outside of the recording range).*

• Regarding the Results: I suggest rewriting this section more accurately. I think it is misleading to say that results show a dissociation between SCR and FPS. This likely happened because the ‘Expectation for Learning’ group failed to show fear acquisition as measured with FPS, thus showing no difference after reinstatement is to be expected. Moreover, authors decided to run an analysis including only participants that showed successful fear acquisition, resulting in no difference among groups. This idea must be rewritten since it is misleading and does not properly convey the results found.

*We have rewritten the beginning of the discussion to more accurately reflect the results, noting that we did find a different pattern of results, but emphasizing that there was no evidence that the expectation for learning enhanced reconsolidation, either for SCR or FPS (page 23-24):*

*The study found a different pattern of results depending on whether SCR or FPS was the unit of measurement. Specifically, for SCR, we found participants across all three conditions (i.e., the expectation for learning, the no expectation for learning, and the no reactivation) exhibited a return of fear following reinstatement on day three of testing. In contrast, FPS remained stable in the expectation for learning group, whereas the no expectation for learning and no reactivation groups showed an increase in FPS to the CS+, suggesting there was a return of fear in the latter two groups. However, this needs to be interpreted in light of the fact that there was no evidence of fear acquisition as measured by FPS, and that the effect size for change in FPS from the end of extinction to start of reextinction in the expectation for learning group was small. Indeed, when looking within only participants that showed evidence of acquisition, FPS remained stable in both reactivation groups, but there was evidence of a return of fear in the no reactivation group. The expectation for learning did not appear to enhance reconsolidation when fear was measured via FPS or SCR.*

• Page 19, Post hoc analysis: authors should specify what post hoc test they decided to conduct.

*We have now included a more detailed description of our post-hoc analyses on page 22-23:*

*Given that not all groups displayed physiological fear acquisition with FPS as the measure of fear, we ran a post hoc analysis including only participants that displayed greater FPS to the CS+ than CS- during the late phase acquisition (n = 20). Though clearly underpowered, we report paired t-tests comparing FPS at the end of day 2 to the start of day 3 for the CS+ and CS- for each group for information purposes.*

• Page 20, Discussion: Authors stated that they found ‘no evidence of reconsolidation’. Reconsolidation takes place every time an individual is shown a reminder of a previously formed memory. What authors could say is instead that the experimental manipulation was not successful in altering this process, thus resulting in the memory being evoked as originally consolidated on day 1. I suggest rephrasing this concept.

*We thank you for this comment. We have reworded this to clarify that it was the manipulation that was not successful, rather than that reconsolidation was not induced throughout the discussion. We have further emphasized the importance of the fact that acquisition was not observed in the entire sample when using FPS as an outcome measure, as a potential explanation of findings.*

• Regarding the Discussion: In my opinion, this paragraph would benefit from some thoughtful as well as in-depth considerations by the authors, because as it stands, it is very descriptive but not enough theoretical as a discussion should be. Authors should make an effort, trying to explain the theoretical implication as well as the translational application of their research. Also, I suggest reorganizing the final part of this section, because I do not think that the results suggest that verbal manipulations of the expectations for learning do not induce reconsolidation, but rather that they are not enough to manipulate said process. Please reshape this section so they can better represent this concept and include the final statements in a ‘Conclusion’ paragraph, to summarize key points and elucidate possible keys to advancing research and understanding of the reconsolidation process.

*We thank the reviewer for these helpful suggestions. We believe that we have addressed the theoretical implications – specifically as they pertain to verbal manipulations of learning expectancy and the outcome variable examined. Given the weak and inconsistent findings with regards to reconsolidation, we are reluctant to comment further, as we believe this would go beyond what we can interpret based upon the current findings. We have included a brief discussion on page 27 concerning the translation implications of our findings, but again, given the non-clinical sample and the fact that we did not observe enhanced reconsolidation with our manipulation, we believe further conjuncture is beyond the scope of our findings. Finally, we have also included a conclusion section highlighting what we believe are the most informative aspects of our findings (29).*

• Although the Authors have acknowledged the study’s limitations at the end of the manuscript, I also suggest including a properly defined ‘Limitations and future directions’ subsection, in which they can describe in detail and report all the technical issues brought to the surface and discuss theoretical and methodological avenues in need of refinement.

*We have consolidated the limitations of the current study into a section with that subtitle (27-29).*

• Regarding the Figures: I suggest adding images of the stimuli and of the experimental design used in the experiment. In my opinion, this visual representation of the experimental procedure will dramatically improve the study's readability and comprehension.

*We have added a figure depicting the main features of our methods (see Figure 1).*

Reviewer #4: Abstract
Objective: the background to the study is not adequately described here

*Thank you for this comment. We have reformulated the abstract to include a brief background description within the confines of the word count limit for the abstract.*

Introduction: In the introduction a lot is written about studies with propranolol, whereby the pharmacological manipulation of the reconsolidation is not the subject of this paper.
The introduction about studies trying to replicate Schillers original results is missing the Paper of Astana et al., (2016), indicating individual differences in Val66Met polymorphism might modulate the effect of reconsolidation.

*We thank you for this suggestion and have included this article in the introduction on page 5.*

Page 5: typing error: ezamined

*This typo has been corrected (see page 5).*

Materials:
The details about the SCR and Starle analyses are totally missing. Please describe in detail the filter, artefact detection approaches, the time windows to define a peak, baseline correction, normalization of the data.
Is it correct, that no participants had to excluded due to missing fear acquisition?

*To clarify, we defined acquisition as being able to accurately report the contingency between the shock and the images at the end of day 1. Participants (n = 6) who were unable to identify the contingency were not invited back for the subsequent sessions. This is outlined on page 13 of the manuscript:*

*At the end of day one, to establish that the shock contingency was learned, participants were asked which image was paired with the shock. Participants were not invited back for the other two days of testing if they were unable to identify the contingency correctly (n = 6).*

*We have also expanded our description of SCR and FPS preparation procedures on pages 11 (for SCR) and 12 (for FPS):*

*For SCR:*

*Data were preprocessed in the EDA analysis software (version 3.1.5) from Mindware Technologies Ltd. We applied a rolling filter with a block size of 100 samples to smooth the data and reduce the influence of noise. All data were visually inspected and rejected when excessive noise distorted the signal (e.g., movement, poor electrode contact). A SCR was defined as a peak minimum of 0.02* $\mu S$ *within 1 to 7 seconds from event onset. SCR corresponds to an increase in* $\mu S$ *amplitude from through to peak, with increases smaller than0.02* $\mu S$ *given a value of 0 .*

*For FPS:*

*The impedance of electrodes was lower than 10* $k\Omega$*.*

*Data were preprocessed in the EMG analysis software (version 3.1.5) from Mindware Technologies Ltd. We segmented data from 0 to 15 seconds post-event onset. We applied a bandpass filter between 28 hz and 500 hz, a notch filter of 60 hz, and a lowpass filter of 5 hz. The data were rectified. All data were visually inspected and rejected when excessive noise distorted the signal, or impedance levels were too high.*

Results
Figure 2 and 4 are not optimal to see the relevant effects.

*We have adjusted Figure 2 and 4 (now Figures 3 and 5) to better demonstrate the relevant effects.*

Literature:
Asthana MK, Brunhuber B, Mühlberger A, Reif A, Schneider S, Herrmann MJ. Preventing the return of fear using reconsolidation update mechanisms depends on the met-allele of the brain derived neurotrophic factor Val66Met polymorphism. International Journal of Neuropsychopharmacology. 2016; 19(6):1–9. DOI: 10.1093/ijnp/pyv137

Reviewer #5: The general approach to use a verbal instruction is interesting regarding potential therapeutic options and to (theoretically) contrast it with pharmacological interventions.
In their point-to-point response the authors addressed the concerns of the reviewers and revised the manuscript substantially. However, reading the manuscript and the responses to the reviewers, I am not convinced that the main concerns we fully addressed. In the current form, the manuscript still lacks some important information or - if given - does not always convey it in an understandable way. There are also several minor language/style/spelling(comma issues that the authors should take care of (to name a few, e.g. “these drugs, are toxic to humans …”, “been replicated [17, 18] however; several …”, “boudnary … ”, “CSa+” in the figure legend).

*The above grammatical issues have been addressed.*

I will state my major concerns in the following. Before that, I would like to say that results are how they are. If something does not speak in favor of a hypothesis, it should be extensively discussed as this improves scientific research.

Major points:
1. Lack of data availability: I am not sure how the Journal handles such issues, although they recommend data to be available to other researchers

*We contacted our research ethics board to inquire as to whether we are able to share the anonymized dataset publicly. Unfortunately, we are not able to make the data set public because participants only consented to only having aggregate data presented. We have included as recommended by the journal information on the means, and standard deviations of each data point presented in supplemental material.*

2. Presentation of procedures/results: The manuscript would benefit a lot from a clearer description of procedures and results. A schematic figure / illustration would be an option. Then, the reader could better grasp the results when having a look at the procedure then.

*Thank you for this comment. We have added a figure with a visual depiction of the major components of the study (see Figure 1).*

3. Related to my second points are some parts of the analysis description that I find hard to understand. For example: “For extinction, the early phase of extinction was calculated by taking the averages of trials one and two on day two. The late phase of extinction was calculated by taking the averages of trials 10 and 11 on day two.” This is one way, but in your specific design, I have some problems to apply this to the three groups. Two groups did 1 CS+ and then after the 10 minutes 10 CS+ and 11 CS-, whereas the third groups did 11 CS+ and 11 CS-. Does this mean that one CS+ of the early extinction process was administered 10 earlier than in the other group (see figures 1 and 3, and page 13, where Extinction trial 1 has only a CS-). In figure 1 or 3: Where is the additional CS+ for the third group? Is it included in the reactivation trial of the other two groups.

*Thank you for requesting clarification on this. We have reviewed and updated the graphs so it clearly indicates the physiological response for the reactivation groups and the no reactivation group for the first presentation of the CS+. As we were reviewing this we also noted a discrepancy in how we computed the first two trials for the CS+ and CS- for SCR on day 2. Though it did not drastically change results, we have updated the results to correct this error (page 19) and have clarified exactly how the early and late phases of extinction are computed in the method section (page 16).*

4. Unwarranted result presentation/conclusions. For example, see the abstract section with the results (“…, and increased in response to the CS+ but not the CS- in the no reactivation group.”). This is actually not the case as this is a result that comes from a posthoc analysis after a non-significant interaction with group. This is not proper statistics. You should only use posthoc analysis if you have significant main and/or interaction effects. Having a closer look at the results shows the following (“The no reactivation group showed an increase in their SCR to the CS+, t (15) = -2.47, p = .03, d = -.84, but not to the CS-, t (15) = -1.86, p = .08, d = -.58, from the end of extinction on day two to the beginning of re-extinction on day three.”). Two things are apparent. First, the phrase “but not to the CS-” results from a p value of .08, with an ES of about .6. The significant comparison “to the CS+” results from a p value of .03, with an ES of about .8. This looks as if one significant posthoc effect and one nonsignificant posthoc effect are interpreted as being different from each other (no interaction).

*Thank you for raising this point. Given the space limitations of the abstract it is difficult to distill the findings to clearly describe all pertinent results. Given the above comments concerning the lack of evidence of fear acquisition using FPS, we have opted to describe the post-hoc findings that include only the sample that showed acquisition. We have made sure to clearly indicate in the abstract that this is post-hoc data. Furthermore, we believe the implications section of the abstract does not overstate the findings as we indicate that there was no evidence that verbal manipulations of learning expectancy enhance reconsolidation. We hoped that this balanced perspective, and clarity concerning post-hoc analyses satisfies the reviewers concerns.*

5. Differences in procedures for the groups. It is suboptimal that there are differences between the groups. For example, on day 2, the third group has a different timing as they did not get a reactivation trial. It might have been better to have a reactivation trial for them, too, but without any verbal instruction. The same is true for the manipulation task, as was already pointed out by the other reviewers. Because of the reactivation for the two groups they have a 10 CS+/11 CS- extinction phase, the third group 11 CS+/11 CS- extinction phase.

*In developing experimental designs, it is indeed a challenge to ensure groups are balanced in every way, particularly for studies using the post-retrieval extinction paradigm. We chose to follow previously established procedures (e.g., Schiller et al., 2010) to make our “reactivation” and “no reactivation” groups as similar as possible. Though reactivation groups differ from the no reactivation group in having one less CS+ presentation DURING extinction, the reactivation and no reactivation groups are EQUIVALENT in the number of presentations of the CS+ and CS- that they received. The difference in timing is what determines whether a group received reactivation or not. Given that this was a key manipulation for the no reactivation group, we have opted not to comment further on this in the manuscript.*

6. Low Number of participants per group and dropouts: This should be properly discussed.

*While it is true that there was a high dropout rate, and we have noted this as a limitation in our manuscript and in a previous reply to our initial revisions (see below), we took steps to ensure that we had adequate power for analyses based on our a priori power analysis, which can be found on page 28 of the manuscript.*

*“This is a good question and something that we wondered as well. We believe the main reasons people dropped out of the study was due to our recruitment method and study schedule. We recruited undergraduate participants from the Integrated System (ISPR) at the University of Ottawa. Participants could only receive two credits total (i.e., one credit for day one and one credit for day two) for their participation in the study. However, participants were required to participant on all three days. Most participants were unaware of the third day of the study requirement (though it was indicated in both the recruitment material and consent form), only required two credits, or had scheduling conflicts which prevented them from participating on all three days. For conciseness we have not included this information in the manuscript, however, we would be happy to do so if the reviewer feels it would be important to add.”*

7. The title should not contain a question. It could also state a null finding.

*We have changed the title to “Verbal manipulations of learning expectancy do not enhance reconsolidation”.*

7. PLOS authors have the option to publish the peer review history of their article ([what does this mean?](https://journals.plos.org/plosone/s/editorial-and-peer-review-process#loc-peer-review-history)). If published, this will include your full peer review and any attached files.

If you choose “no”, your identity will remain anonymous but your review may still be made public.

**Do you want your identity to be public for this peer review?** For information about this choice, including consent withdrawal, please see our [Privacy Policy](https://www.plos.org/privacy-policy).

Reviewer #3: No

Reviewer #4: No

Reviewer #5: No
